# Nanoencapsulation of Methylene-Blue for Enhanced Skin Cancer Cell Phototoxicity and Cutaneous Penetration in Association with Sonophoresis

**DOI:** 10.3390/pharmaceutics15051371

**Published:** 2023-04-29

**Authors:** Thayane Soares Lima, Monalisa Sthefani Silva de Oliveira, Alice Vitoria Frota Reis, Raquel Petrilli, Josimar O. Eloy

**Affiliations:** 1 Department of Pharmacy, Dentistry and Nursing, Faculty of Pharmacy, Federal University of Ceará, 1210 Pastor Samuel Munguba Street, Fortaleza 60430-160, CE, Brazil; thayanelima67@gmail.com (T.S.L.); monalisafarmacia@alu.ufc.br (M.S.S.d.O.); avfr_frota@hotmail.com (A.V.F.R.); 2 Institute of Health Sciences, University of International Integration of the Afro-Brazilian Lusophony, Redenção 62790-000, CE, Brazil; petrilliraquel@unilab.edu.br

**Keywords:** methylene blue, nanoparticles, photodynamic therapy, skin neoplasms, skin absorption, phonophoresis

## Abstract

Photodynamic therapy (PDT) using methylene blue (MB) as a photosensitizer has emerged as an alternative treatment for skin cancers, such as squamous cell carcinoma (SCC). To increase the cutaneous penetration of the drug, some strategies are used, such as the association of nanocarriers and physical methods. Thus, herein we address the development of nanoparticles based on poly-Ɛ-caprolactone (PCL), optimized with the Box–Behnken factorial design, for topical application of MB associated with sonophoresis. The MB-nanoparticles were developed using the double emulsification-solvent evaporation technique and the optimized formulation resulted in an average size of 156.93 ± 8.27 nm, a polydispersion index of 0.11 ± 0.05, encapsulation efficiency of 94.22 ± 2.19% and zeta potential of −10.08 ± 1.12 mV. Morphological evaluation by scanning electron microscopy showed spherical nanoparticles. In vitro release studies show an initial burst compatible with the first-order mathematical model. The nanoparticle showed satisfactory generation of reactive oxygen species. The MTT assay was used to assess cytotoxicity and IC_50_; values of 79.84; 40.46; 22.37; 9.90 µM were obtained, respectively, for the MB-solution and the MB-nanoparticle without and with light irradiation after 2 h of incubation. Analysis using confocal microscopy showed high cellular uptake for the MB-nanoparticle. With regard to skin penetration, a higher concentration of MB was observed in the epidermis + dermis, corresponding to 9.81, 5.27 μg/cm^2^ in passive penetration and 24.31 and 23.81 μg/cm^2^ after sonophoresis, for solution-MB and nanoparticle-MB, respectively. To the best of our knowledge, this is the first report of MB encapsulation in PCL nanoparticles for application in skin cancer using PDT.

## 1. Introduction

Skin cancer is one of the most common types of cancer worldwide. Its high prevalence and incidence makes it an important health problem [1]. The most common category of skin cancer is those classified as non-melanoma type (NMSCs), being subdivided into basal cell carcinoma (BCC), which forms in basal cells, and squamous cell carcinoma (SCC), which originates from rapidly proliferating malignant cells in the epidermis. SCC has attracted attention in recent years for its aggressive potential to cause metastasis to distant sites or lymph nodes and the significant increase in its rate of occurrence since 1976–2010 [2]. Risk factors for its development arise from a combination of environmental and genetic factors, the most common cause being prolonged exposure to ultraviolet (UV) light. Traditionally, the first-line treatment for NMSCs has been surgical modalities such as excision, Mohs micrographic surgery, and electrode-desiccation with curettage. However, this therapy is dependent on several variables such as the patient’s comorbidities, size of the affected area, multiplicity of lesions and even the possibility of formation of large post-surgical scars. Within this context, there is great relevance in the search for new therapies aimed at improving the quality of treatment for patients [3,4].

Historically, photodynamic therapy (PDT) has been used to treat a variety of diseases [5]; however, its minimally invasive treatment characteristics, combined with application specificity and low cumulative toxicity, proved to be suitable for an efficient alternative approach to treat or limit resistant cancers. In addition, PDT, by using the topical route for the application of its photosensitizer, manages to add advantages such as the decrease in side effects and the increase in the localized concentration of the drug in the layers of the skin. Its mechanism of action is based on the activation of a photosensitizing agent by light at a specific wavelength, resulting in the generation of reactive oxygen species (ROS), mainly singlet oxygen, which cause damage to cellular components resulting in cancer cell death [6]. Methylene blue (MB), a dye belonging to the phenothiazine family, is one of the most commonly used photosensitizers due to its high generation of ROS and healing properties [7]. In the context of cancerous diseases, MB has already been used in breast cancer [8], melanoma [9], lung cancer [10] and in the SCC itself [11]. Taken together, based on in vitro, in vivo and preclinical studies, MB has proven to be a promising agent that can be used for the treatment of SCC [11,12,13].

Since the topical delivery of the photosensitizer seems to be one of the most important stages of PDT, there is a need to ensure that its physicochemical characteristics will be preserved, in addition to favorable pharmacokinetics, the high supply of singlet oxygen it is also important to prevent drug aggregation. Furthermore, the treatment should not have side effects that could be harmful to normal cells [14]. Another relevant point is the skin barrier. It is common knowledge that it is the largest organ in the human body serving as the first line of defense for microorganisms and foreign substances. Its most superficial layer, the epidermis, performs this fundamental role through the stratum corneum (SC). The SC is a thick matrix of keratinocytes interspersed with lipids, so hydrophilic molecules of high molecular weight are difficult to penetrate. Faced with both physical-chemical and biological problems, the development of new drug delivery systems to preserve their characteristics and improve penetration into the skin is highly recommended [3].

In this context, polymeric nanocarriers have been a rising platform for targeted application to cancer. These nanosystems have advantages of good drug transport capacity, controllable particle size, biodegradability, in addition to the adjustment in the release profile and effective targeting. In addition, the double emulsification and solvent evaporation method is suitable for encapsulation of hydrophilic molecules such as MB. It is known that the encapsulation of small hydrophilic molecules remains a challenge, due to their good solubility in water and low molecular weight, MB can have weak interactions with the polymeric structures that form the nanoparticles, causing low encapsulation efficiency, unwanted leakage and a rapid initial release [15,16]. Even with these obstacles, MB has already been successfully encapsulated within polymeric matrices [17,18,19].

In cutaneous delivery of drugs, polymeric nanoparticles could accumulate in hair follicles forming a drug reservoir providing sustained/controlled release [20]. Several studies have already used these polymeric platforms and reported improvement in both the most superficial and deepest layers of the skin [21,22]. Still, to enhance the delivery of topical nanoparticles, physical methods are extensively used to destabilize the SC. Sonophoresis is a technique that uses ultrasound, which can be of low or high frequency, in contact with the skin, being a minimally invasive pre-treatment [23]. Herein, we show for the first time the use of MB-containing PCL nanoparticles associated with sonophoresis for PDT of skin cancer. More importantly, in the present study, we hypothesized that sonophoresis can increase the cutaneous penetration of MB-loaded polymeric nanoparticle.

In an effort to improve the effects of PDT by methylene blue, this study synthesized polycaprolactone (PCL) nanoparticles that were optimized by Box–Behnken design and characterized for size, encapsulation efficiency, release profile, reactive oxygen species generation, photocytotoxicity and cellular uptake. Furthermore, a skin penetration study was proposed to investigate the association between sonophoresis and nanoparticle as a more effective approach for better cutaneous delivery of MB.

## 2. Materials and Methods

### 2.1. Material

Methanol was obtained from J.T. Baker (Phillipsburg, PA, USA) and dichloromethane (DCM) from NEON (Suzano, SP, Brazil). Polyvinyl alcohol, methylene blue, poly-ε-caprolactone (PCL), rhodamine 123, 3-[4,5-dimethyl-thiazol-2-yl]-2,5-diphenyltetrazolium bromide (MTT), dimethyl sulfoxide (DMSO), 4′,6′-diamino-2-phenylindole (DAPI), Dulbecco’s Modified Eagle’s Medium (DMEM), trypsin, Fluoromount^®^ mounting medium, antibiotic/antimycotic solution, paraformaldehyde were obtained from Sigma-Aldrich (St. Louis, MO, USA). Potassium chloride (KCl) was obtained by Synth (Diadema, SP, Brazil). PTFE filters (25 mm, 0.45 μm, Allcrom^®^, São Paulo, SP, Brazil) were obtained from Allcrom^®^. The human cell line model of squamous cell carcinoma A431 was obtained from the Cell Bank of Rio de Janeiro (Brazil).

### 2.2. Development of Nanoparticles and Factorial Design

The preparation of NPs followed the classic technique of double emulsification and solvent evaporation previously described [24]. First, 2 mg of MB was dissolved in 2 mL of aqueous solution containing polyvinyl alcohol (PVA) and then added to a dichloromethane (DCM) solution containing the polycaprolactone. Thereafter, the mixture was emulsified by sonication with probe ultrasound (QSonica Sonicators Q500, Newtown, CT, USA) for 3 min at 30% amplitude under an ice bath. Soon after, the primary emulsion was dropped to 25 mL of the 1% PVA solution and then re-emulsified under the same sonication conditions as before. Finally, DCM was evaporated under magnetic stirring at room temperature in chemical hood for 1 h. The emulsification steps were monitored by optical microscopy.

To check and optimize the response parameters of the formulations, the Box–Behnken design method was used using the Minitab^®^ 19.0 software (Minitab, Philadelphia, PA, USA). The BBD presented three factors at three levels (high, medium, and low) that generated second-order polynomial equations and response surface graphs. With this, the relationship between the independent variables was investigated: organic phase/aqueous phase ratio of the 1st emulsification (X_1_), percentage of PVA in the 1st emulsification (X_2_) and amount of polycaprolactone (X_3_) and their impacts on the dependent variables: particle size (Y_1_), polydispersion index (Y_2_) and encapsulation percentage (Y_3_) of the formulations. The generated polynomial model consists of:

(1) $$Yi=B_{0}+B_{1}X_{1}+B_{2}X_{2}+B_{3}X_{3}+B_{12}X_{1}+B_{13}X_{1}X_{3}+B_{23}X_{2}X_{3}+B_{11}X_{1}^{2}+B_{22}X_{2}^{2}+B_{33}X_{3}^{2}$$

wherein Y_i_ represents the dependent variable; B_0_ the interception, X_1_, X_2_ e X_3_ the independent variables and B_1_ a B_33_ their respective regression coefficients.

Thus, the optimization was performed through the desirability approach, in which the analysis of the independent variables of 15 formulations aimed to achieve a smaller particle size and PDI, together with a greater encapsulation efficiency.

### 2.3. Physical-Chemical Characterization

#### 2.3.1. Particle Size, Polydispersity Index (PdI), and Zeta Potential Analysis

Aliquots of nanoparticles (100 μL) were diluted in 900 μL of potassium chloride (KCl) solution 1 M and analyzed in cells with 1 cm of optical path using dynamic light scattering (DLS) in the Zetasizer device Nano ZS (Malvern Instruments) at 25 °C for particle size and PdI measurements. The zeta potential was determined in the same equipment with appropriate cuvettes, based on the electrophoretic mobility of dispersed particles when subjected to an electric field.

#### 2.3.2. MB Encapsulation Efficiency (EE%)

For the calculation of encapsulation efficiency, the indirect method described by Crisóstomo et al. was used with adaptations [25]. Herein, the amount of free drug is calculated and correlated with its theoretical amount. For this, it is necessary to centrifuge an aliquot of the formulation (1 mL) in an Amicon^®^ 50 kDa tube at 3000× *g* for 10 min. Afterwards, the centrifuge is diluted in distilled water (1:10) and read in a spectrophotometer at a wavelength of 570 nm. The absorbance results obtained were plotted on the straight-line equation resulting from the drug calibration curve, for quantification of the free portion. The calculation of the encapsulation percentage is then represented by Equation (2):

(2) $$EE\%=\frac{\left[MB\right]total-\left[MB\right]free}{[MB]total}\times 100$$

where [MB]total represents the total theoretical concentration of MB in the nanoparticles and [MB]free represents the free drug concentration.

#### 2.3.3. Morphology Evaluation by Scanning Electron Microscopy

The mean diameter and morphology of the NPs were examined by scanning electron microscopy (SEM) using a Scanning Electron Microscope Quanta 450 FEG-FEI, with a nominal resolution of 1 nm. Aliquots of the formulation were placed on an aluminum sample mounter, on a carbon tape, dried at room temperature and spray-coated with gold, in order to increase the surface conductivity. SEM images were captured at 150,000× and 10 kV.

### 2.4. In Vitro Drug Release

The in vitro release was evaluated by comparing the methylene blue solution with the nanoparticle formulation, using the passive release method. Thus, 1 mL of the samples were placed in contact with 7 mL of PBS pH 7.4 at 37 °C in sealed beakers and kept under constant agitation in an orbital shaking incubator at 150 rpm. The experiments were conducted in quadruplicate with independent samples for each collection time. Every 1, 3, 5, 8, 10, 24 h, 2 mL of each sample was placed in an Amicon^®^ 50 kDa tube and centrifuged for 10 min at 4000× *g*. The filtrate was diluted with distilled water (1:3) and read in a spectrophotometer at 663 nm. The percentage of MB released from each sample is graphically represented as a function of time to evaluate the drug release profile. In addition, the release kinetics was evaluated (DDSOLVER), submitting the data obtained in the test to zero order kinetics (Equation (3)), first order kinetics (Equation (4)), the following models were also used: Higuchi (Equation (5)), Weibull (Equation (6)) and Hopfenberg (Equation (7)). Observing the linear correlation coefficients between the models, based on their respective equations below:

(3) $$\mathrm{Zero}\;\mathrm{order}\;\mathrm{model}:\;M_{T}=M_{B}+K_{0}t.$$

(4) $$\mathrm{First}\;\mathrm{order}\;\mathrm{model}:\;ln(M_{0}-M_{T})-ln(M_{0})-K_{1}t.$$

(5) $$\mathrm{Higuchi}’s\;\mathrm{model}:\;mt=K_{H}t^{0.5}.$$

(6) $$\mathrm{Weibull}’s\;\mathrm{model}:\;F=100\cdot [1-e^{-\frac{\left(t-Ti\right)\beta }{a}}].$$

(7) $$\mathrm{Hopfenberg}\prime s\;\mathrm{model}:\;F=100\cdot \left[1-{\left(1K_{HC}.t\right)}^{3}\right]$$

### 2.5. Reactive Oxygen Species (ROS) Generation

1,3-diphenylisobenzofuran (DPBF) was used as an indicator to detect ROS production by methylene blue in solution or encapsulated in polymeric nanoparticles. Briefly, 200 uL of the solution (240 µg/mL in DMSO) and methylene blue nanoparticle (80 µg/mL) were placed in a 96-well plate and added with 100 uL of DPBF solution (final concentration: 30 µM in DMSO) under dark conditions. Immediately, representing time 0, the absorbance of the samples at 410 nm was recorded using a microplate reader. Soon after, the plate was subjected to light irradiation (630 nm, 6.7 J/cm^2^, 0.001841 W/cm^2^) for 3 min followed by absorbance recording until 30 min of irradiation. Control groups of DPBF, DMSO and nanoparticles without methylene blue were used to remove possible interferences [9,26].

### 2.6. Cell Studies

#### 2.6.1. Cell Culture

To evaluate the photocytotoxicity and cell uptake of MB-loaded nanoparticle, a human cell line (squamous cell carcinoma) A431 was used. The cells were cultured in Dulbecco’s Modified Eagle’s Medium (DMEM) supplemented with 10% fetal bovine serum and 1% antibiotic/antimycotic solution, at 37 °C with 5% CO_2_, according to the recommendations of the American Type Culture Collection (ATCC).

#### 2.6.2. Photocytotoxicity and Cytotoxicity

The photocytotoxicity assay was evaluated by the MTT method. Cells from the suspension were seeded into 96-well plates (1 × 10^4^ cells/well) and left for fixation for 24 h at 37 °C. After that, they were washed with saline solution and the treatments (methylene blue solution, methylene blue nanoparticle and white nanoparticle) applied in concentrations of 0.05; 0.5; 5; 12.5; 25 and 50 µM. After applying the treatment, the plates were incubated for 5 min or 2 h (pre-irradiation times) in an incubator at 37 °C and then cells were exposed to red laser illumination (630 nm, 6.7 J/cm^2^, 0.001841 W/cm^2^) for 60 min. After 1 h, the treatments were removed, the wells washed and incomplete DMEM medium applied and the plates again incubated for 24 h at 37 °C. Subsequently, 170 µL of incomplete culture medium was applied together with 30 µL of MTT solution (250 µg/mL), followed by incubation (4 h at 37 °C). Subsequently, after discarding the MTT culture medium solution, dimethyl sulfoxide (DMSO) was applied to the wells to dissolve the formazan crystals. The absorbance was read at 562 nm and the concentration able to kill 50% of the cells (IC50) was obtained through the concentration-effect curves, considering the optical density of the negative control (untreated cells) as 100%. Finally, cytotoxicity in the dark was evaluated in the same way for the treatments groups without light irradiation [27]. The temperature was monitored during the irradiation period.

#### 2.6.3. Cellular Uptake

Photomicrographs were recorded by fluorescence confocal microscope (LSM 700, Zeiss) to evaluate nanoparticle uptake by the A431 cell line. The nanoparticle formulation was prepared without MB and with the mitochondrial fluorescent marker, Rhodamine 123 (40 µg/mL). Thus, for confocal studies, 5 × 10^5^ cells/well were applied to sterile 22 mm/22 mm coverslips placed in 6-well microplates and incubated for 24 h under the same conditions mentioned above. After cell adhesion, protected from light, 1900 μL of incomplete medium was inoculated and 100 μL of nanoparticle samples were added for incubation for 1, 3, 6 or 24 h at 37 °C with an atmosphere of 5% CO_2_.

After incubation, the wells were washed three times with 0.9% saline solution (1 mL), and then the cells fixed with 1% paraformaldehyde (2 mL) for 15 min. After this time, three washes were performed with 0.9% saline solution for subsequent addition of 900 μL of DAPI solution (0.3 μg/mL) for staining the cell nucleus. The coverslips were washed again and, in order to preserve the fluorescence, they were placed on a histological slide containing Fluoromount^®^ mounting medium. We used λ_ex_ = 488 nm and λ_em_ = 575 nm for Rhodamine 123 and λ_ex_ = 340 nm and λ_em_ = 488 nm for DAPI. Images were processed using Zeiss ZEN Blue Edition 3.4 software [28].

### 2.7. In Vitro Skin Penetration Studies

#### 2.7.1. Quantification of MB in Porcine Ear Skin by UV-Vis Spectrophotometry

To quantify MB in skin layers, a previously developed method was employed with modifications [29]. Pieces of porcine ear skin obtained from a local slaughterhouse (Fortaleza, Ceará), with an area of 0.95 cm^2^, were used to develop a calibration matrix-based in triplicate. In recovery studies, the skin was contaminated with known concentrations of MB and after drying the solution, extraction was performed using methanol. In this process, the samples were subjected to vortex mixing for 2 min and then placed in an ultraturrax for 3 min at 7000 rpm. To enhance the extraction, the samples were placed in an ultrasound bath for 45 min and centrifuged at 4000 rpm for 20 min. Finally, they were filtered with a 0.45 µm PTFE filter. For the quantification of the samples, UV-Vis spectrophotometry was used with reading in the range of 663 nm. In order to analyze the interference of skin components in the spectrophotometric method, a spectral scan was carried out following the same methodology without application of methylene blue mother solution. The recovery calculation was performed using the absorbance of MB extracted from the skin in relation to the absorbance of MB in methanol. The results were presented in percentage of recovery.

#### 2.7.2. Quantification of MB in the Skin Layers and in the Receptor Compartment

The skin of porcine ears was carefully dissected with a scalpel and the subcutaneous adipose tissue was removed with surgical scissors. Skin cuts with any wounds, bleeding, skin diseases, cuts or holes in the surface were discarded. An area of approximately 0.95 cm^2^ was cut and mounted in the Franz cell apparatus (*n* = 5) with the stratum corneum facing the donor compartment. Skin resistivity was calculated according to topic 2.6.3.1 and only samples with initial resistivity greater than 35 kΩ cm^2^ were used [30]. MB-solution and MB-nanoparticle (80 μg/mL) were added to the donor compartment (1mL) and the receptor compartment was filled with 15 mL of PBS (pH 7.4). The assembled Franz cells were left under magnetic stirring at 150 rpm for 16 h at 32 °C.

At the end of the experiment, the apparatus was disassembled, and the skins were superficially washed with distilled water and dried. For extraction of the MB in the stratum corneum, the tape stripping protocol was used with Scotch Book Tape n° 845, 3 M. With that, 15 pieces of adhesive tape were adhered to the stratum corneum and removed immediately afterwards, always discarding the first tape, while the others were placed in a falcon tube containing 5 mL of methanol. For extraction of MB in the viable epidermis and dermis, the skin remaining from the tape stripping was used, cut into small pieces and placed in a falcon tube containing 5 mL of methanol. Finally, the PBS present in the receptor compartment was also collected.

For quantification, all samples were vortexed for 2 min. Viable epidermis + dermis samples followed additional steps in ultraturrax for 3 min at 7000 rpm, ultrasound bath for 45 min and centrifugation at 4000 rpm for 20 min, in that order, respectively. Finally, all samples were filtered through a 0.45 µm PTFE filter and stored to be quantified as described in topic 2.6.1.

#### 2.7.3. Pre-Treatment of the Skin with Sonophoresis

##### Measurements of Electrical Conductivity of the Skin

Skin integrity and the effect of ultrasound pre-treatment were assessed by measuring skin resistivity before and after sonophoresis. For this, 4.0 mm Ag/AgCl disk electrodes were introduced into the donor and receptor compartment of the Franz diffusion cells, filled with PBS (pH 7.4). A power of 100 mV (RMS) and 10 Hz frequency was adopted using a signal generator equipment and an alternating current was applied. The intensity of the electric current able to cross the skin was measured using a multimeter. Skin resistivity was calculated using Ohm’s law multiplied by the area of exposed skin (0.95 cm^2^), aiming for values around 1 kΩ cm^2^ after sonophoresis [31].

##### Ultrasound Application

The experiments were carried out in Franz cells mounted with porcine ear skin as described above. The donor compartment was filled with 2 mL of PBS containing 1% SDS as a coupling medium while the receptor compartment was filled with 15 mL of PBS (pH 7.4). The ultrasound (QSonica Sonicators Q500, operating at a frequency of 20 kHz equipped with a 13 mm diameter probe) was positioned in the donor compartment, at 5 mm from the skin surface. The exposure time was 1 min, with a pulse of 5 s “on” and 5 s “off” and amplitude of 20%, until the skin resistivity of 1 kΩ cm^2^ was reached. This step was monitored in order to avoid an abrupt rise in temperature. After sonication, the skin resistivity was measured, and the contents of the recipient and donor compartments were replaced to continue the evaluation of MB penetration into the skin layers after application of sonophoresis.

### 2.8. Statistical Analysis

In vitro release was analyzed by two-way ANOVA test with Bonferroni post-test in GraphPad Prism software. The in vitro results of cytotoxicity and skin penetration were analyzed by one-way ANOVA, with post hoc Bonferroni test in the same software. Data were estimated as a mean ± standard deviation and statistical differences (* *p* < 0.05) of comparisons of means are reported.

## 3. Results and Discussion

### 3.1. Factorial Design and Physicochemical Characterization of Nanoparticles

The delivery of hydrophilic substances, such as MB, is challenging, especially when focused on topical application, because the hydrophilic nature prevents the arrival of drugs in deeper layers of the skin. Furthermore, hydrophilic drugs are also difficult to encapsulate. In this context, polymeric nanoparticles have been widely used for drug encapsulation, with several advantages, including protection against degradation, sustained release and improved skin delivery. Double emulsion and solvent evaporation is a widely used method for encapsulating hydrophilic molecules [24]. It is evident, however, that several factors can affect this encapsulation, including the ratio between the organic and aqueous phases, molecular weight and amount of polymer and the use of surfactants. Herein, the Box–Behnken Design (BBD) aimed to obtain an optimized formulation, establishing relationships between the responses and a set of parameters that can interfere directly or indirectly with a minimum number of experimental runs [16,32].

Table 1 presents the results of the interactions between the independent variables: organic phase/aqueous phase ratio of the 1st emulsification (X_1_), percentage of polyvinyl alcohol (PVA) of the 1st emulsification (X_2_) and amount of polycaprolactone (X_3_) and the impacts on the dependent variables: particle size (Y_1_), polydispersion index (Y_2_) and encapsulation efficiency (Y_3_), in addition to the zeta potential results. The minimum and maximum values for Y_1,_ Y_2,_ Y_3_ comprise between 160–278.06 nm, 0.097–0.3323 and 94.37–99.44%, respectively.

**Figure 1:**
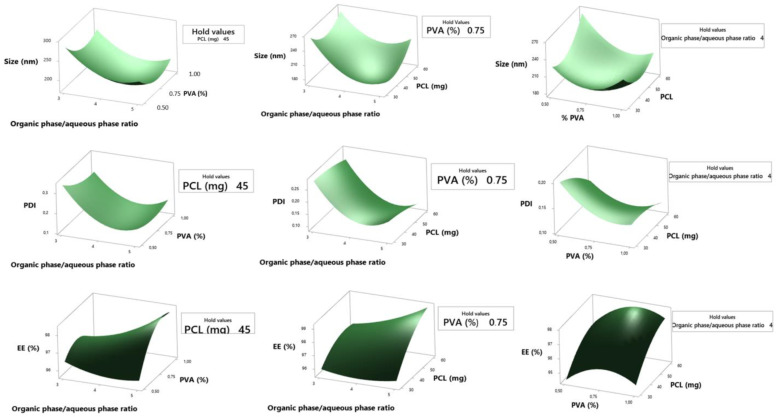
Graphs of surface response of MB-loaded nanoparticles using organic phase/aqueous phase ratio of the first emulsification (X_1_), polyvinyl alcohol percentage of the first emulsification (X_2_ and amount of polycaprolactone (X_3_) as independent variables.

For a better understanding of the influence of the independent variables and to study the effects of each factor and their interaction on the responses considered, the present study used a multiple regression that generated a second-order polynomial equation in a full quadratic model and response surface graphs, as shown in Equations (8)–(10) and Figure 1.

(8) $$Y_{1}=1327-323.0X_{1}-628X_{2}-9.00X_{3}+13.4X_{1}X_{2}+0.628X_{1}X_{3}-3.10X_{2}X_{3}+32.2X_{1}^{2}+443X_{2}^{2}+0.1035X_{3}^{2}.$$

The equations obtained allow us to draw conclusions through the magnitude of the coefficients and their mathematical sign. A positive sign means a synergistic effect, a negative sign means an antagonistic effect. The X_1_, X_2_ e X_3_ represents the results obtained for changing one variable at a time. X_1_X_2_, X_1_X_3,_ X_2_X_3_, X_1_^2^, X_2_^2^ e X_3_^2^ represent their interactions simultaneously. For the particle size the interaction between the terms X_1_X_2_, X_1_X_3_, X_1_^2^, X_2_^2^ e X_3_^2^, seems to denote a favorable effect in obtaining the desired response. Based on the values of the correlation coefficient (R^2^) there was a satisfactory fit of the regression to the particle size response model with a value of 0.8890.

From Equation (8) and Figure 1, it is evident that, by increasing the ratio of the organic phase/aqueous phase together with the percentage of PVA, the size of the nanoparticles decreases. This can be explained primarily by the fact that the addition of surfactants in emulsions plays a key role in their stability, increasing their surface coating, forming a protective film around the droplets, leading to the formation of smaller sizes and preventing their coalescence. In the solvent evaporation step, these characteristics become important, as the emulsion volume can decrease, consequently increasing its viscosity, leaving the final droplet size larger, resulting in a larger NP. Using the same synthesis methodology, Iqbal et al., 2015 found similar behaviors with respect to PVA when developing PCL nanoparticles. In their study, it was observed that the size of the nanoparticles decreased when increasing the PVA concentration from 0.05 to 0.2% [24]. Furthermore, a greater volume of organic solvent to dissolve the polymer prevents the formation of a viscous primary emulsion, resulting in a more effective reduction in particle size during the second step of the emulsification process.

The polydispersion index (PDI) indicates the homogeneity of the nanoparticle size groups. A PDI value close to 0 means that the system has groups that indicate a narrow size distribution, whereas close to 1 a wide size distribution. In Equation (9), it is noted that the isolated term X_3_ and your interaction X_2_X_3_ in addition to the interaction between X_1_X_2_, X_1_^2^, X_2_^2^, favor the answer, although the isolated terms X_1_ e X_2_ are antagonistic. The mathematical model had a satisfactory fit showing R^2^ of 0.9029.

(9) $$Y_{2}=2.189-0.731X_{1}-1.113X_{2}+0.00035X_{3}+0.0803X_{1}X_{2}-0.00018X_{1}X_{3}+0.0028X_{2}X_{3}+0.0770X_{1}^{2}+0.39X_{2}^{2}-0.000034X_{3}^{2}.$$

The results presented in Figure 1 reveal that, as well as for the particle size, the ratio between the organic phase/aqueous phase and its relationship with PVA seems to have positive impacts for the reduction of the PDI. Likewise, increasing surfactant concentration can lead to the formation of nanoparticles of uniform size, resulting in narrower PDI ranges. In this study, an average PDI of 0.18 was reached, which is in agreement with Shaikh, Kala and Nivsarkar who found a variation from 0.018 to 0.192 when optimizing PLGA nanoparticles loaded with Doxorubicin [33].

With regard to MB entrapment in NPs, the results presented in Table 1 showed that the encapsulation efficiency was high, an average of 96.61%. The polynomial Equation (10) shows the effect of the independent variables, where it is observed that the isolated term X_1_, and its interaction with X_3_, besides X_2_^2^ and X_3_^2^, have negative effects on the response. The mathematical model obtained a regular fit with R^2^ of 0.6305.

(10) $$Y_{3}=99-5.67X_{1}+10.2X_{2}+0.113X_{3}+2.93X_{1}X_{2}-0.0352X_{1}X_{3}+0.039X_{2}X_{3}+0.3X_{1}^{2}-14.8X_{2}^{2}-0.00234X_{3}^{2}.$$

According to Figure 1, a relationship between the percentage of PVA and the amount of PCL is suggested, where values of 0.8 to 0.9% of surfactant and 50 to 60 mg of polymer result in greater drug entrapment. One of the factors that can explain the high encapsulation is the structure of the nanoparticle, with a probable formation of nanocapsules in the synthesis process. With a defined core-shell structure, the entrapment properties of the drug within the core are increased. Methylene blue, in turn, solubilized in the internal aqueous phase and was coated by the polymer layer, which protects the drug from mechanical, physical and chemical factors that could degrade it. Furthermore, studies claim that the dual solvent emulsification-evaporation method has an average drug encapsulation efficiency of 65% to 75%, which could corroborate this study [34]. Finally, a previous study obtained 56.2 ± 3.2% MB encapsulation in calcium phosphate nanoparticles with application in photodynamic therapy [35].

The zeta potential is a parameter that provides information about the electrostatic potential of particles in solution and describes their stability in a colloidal system. Particles with values greater than +30 mV/−30 mV are considered stable [36]. However, these values are subject to change depending on the physicochemical characteristics of the components used in the preparation of NPs, in addition to the pH, concentration, ionic strength of the solution and the nature of the surface binders. In this study, the MB-nanoparticles obtained negative values in all measurements with an average of −4.73 mV, mainly due to the presence of terminal carboxyl groups existing in the PCL. This negative trend of the zeta potential when using PCL has already been reported in studies such as the one conducted by Badri et al., in which the values found ranged from −6.51 mV to −7.0 mV. In this study, the authors also noted an inversely proportional relationship between PVA concentration and zeta potential, in which there was a decrease from 7.38 mV to 4.45 mV when increasing the concentration of surfactant from 2.5 mg/ml to 20.0 mg/ml, which may explain why the zeta potential of the MB-nanoparticle does not exceed values greater than −30 mV [30]. Although the zeta potential provides clues about the stability of the colloid, it is not the only factor responsible. The stability of the system depends on the attractive forces of van der Waals and electrostatic repulsive forces, the zeta potential provides only the last mentioned, being common to find stable colloids with low values [37,38].

The optimized formulation of MB-loaded NPs was predicted using the desirability approach in the software. The objective was to formulate NPs with minimum particle size and PDI, together with maximum EE%. Table 2 shows the actual optimized values of the levels X_1_, X_2_ e X_3_, the estimated and obtained results for each answer and the respective relative errors. The result for desirability was 0.8115, which indicates adequacy of the mathematical models for the experimental data, since the scale varies between d = 0 (for an unacceptable response value) and d = 1 (for a completely desirable value). In general, the measured values obtained were satisfactory with the values calculated from the Box–Behnken design. The PDI (Y_2_) showed the most adequate fit with a relative error of 2.56%, while the particle size (Y_1_) managed to obtain even better values than expected. Thus, the formulation proved to be suitable for future studies.

#### Morphology and Particle Size Distribution of Nanoparticles

**Figure 2:**
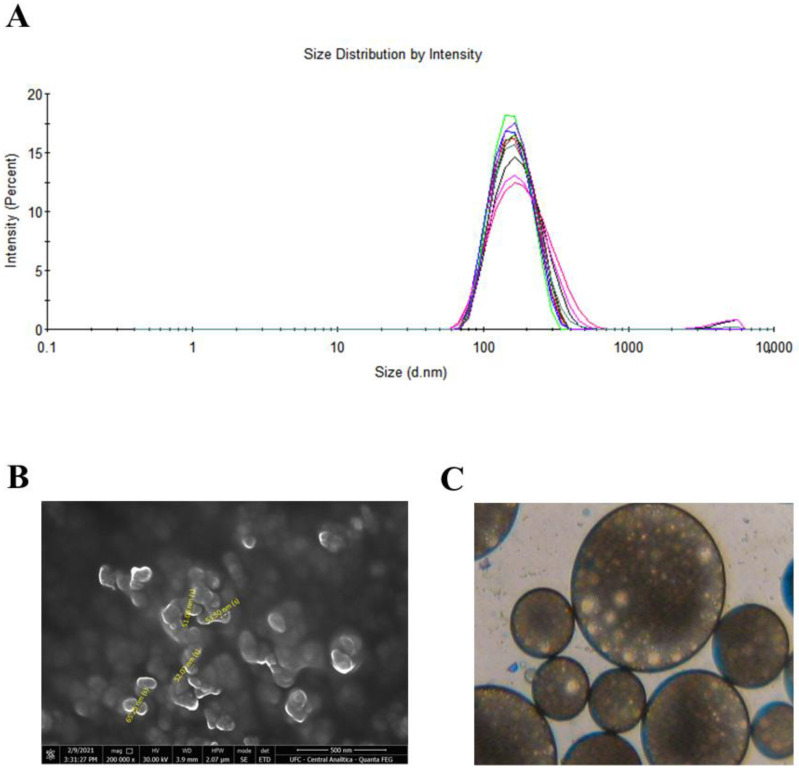
(**A**) Particle size distribution graph using DLS (**B**) Scanning electron microscopy image of nanoparticles containing MB (**C**) Optical microscopy observation of the double emulsion during the preparation process of the optimized formulation.

NPs were investigated with respect to shape, surface morphology and size by SEM. According to Figure 2, the spherical shape was observed as the predominant morphology and particle size was smaller than 100 nm. It was noted, however, that the size values found in SEM are smaller than those reported in the particle distribution graph, which may occur due to the aggregation of the particles in the DLS analysis. Furthermore, it is expected that the size obtained by DLS is larger in comparison with SEM, since the first provides the hydrodynamic diameter and the second the actual size [38]. For the topical delivery of drugs, it is important that the NP be of small size, promoting a larger surface area in contact with the skin, facilitating the cutaneous penetration of therapeutic agents [39]. It was also possible to monitor the formation of the double emulsion (W/O/W) in the step before solvent evaporation. Finally, it can be stated the formation of polymeric nanoparticles was successful.

### 3.2. In Vitro Drug Release Study

**Figure 3:**
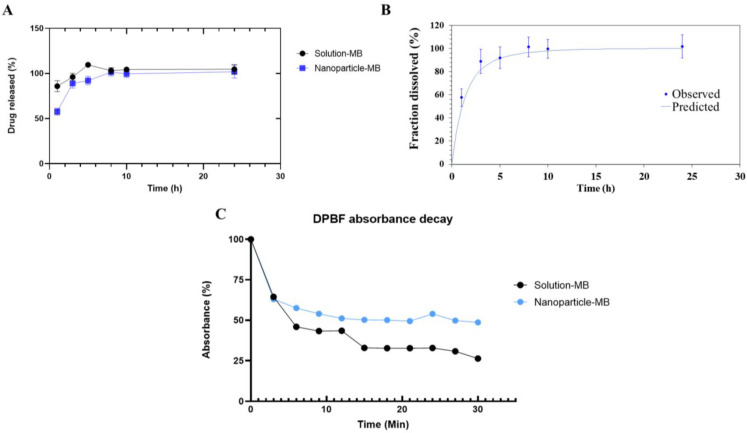
(**A**) In vitro release profile comparing solution and MB nanoparticle (**B**) Graph contemplating the modeling of nanoparticle dissolution data in the first-order model (**C**) Study of the decay of DPBF absorbance in the presence of the solution and methylene blue nanoparticle, with irradiation (630 nm, 6.7 J/cm^2^, 0.001841 W/cm^2^) in time intervals of 3 min, until 30 min of light irradiation.

The in vitro release study aimed to compare the release profiles of aqueous methylene blue solution and the optimized formulation of the nanoparticle. The release mechanism is characterized by an initial burst, that is, a rapid release of the drug into the medium, both for the solution group and for the nanoparticle. Although in vitro release studies with methylene blue nanoparticles are scarce in the literature, Gutiérrez-Valenzuela et al., working with various AM concentrations, already demonstrated that the release profile of this drug tends to be faster. As shown in Figure 3A, in the first hour, an average release of 85.88% is observed for the solution, thus indicating that the release of MB by the solution is almost immediate, due to the hydrophilic nature of the drug. The nanoparticle is able to trap the drug a little more effectively, with drug release values of 57.67%. Based on the hydrophilicity of MB and its small size, it is expected that it can “escape” from the nanoparticle and accumulate in the aqueous phase of the formulation, increasing its initial burst release. Another hypothesis would be the occurrence of nanoencapsulation close to the surface of NPs. After that, between the interval of 8 to 24 h, a plateau was observed, possibly indicating that all the MB was released, for both groups. This can be explained by the diffusion of MB through the polymer that composes the nanoparticle [19]. For the solution, an increase in the values of the percentage of MB released as a function of time was observed, with a slight decay after the 5 h point. It was possible to observe a statistically significant difference between the solution and the nanoparticle at times of 1 h and 5 h (*p* < 0.05), thus emphasizing that although the nanoparticle also has a pronounced release, it is still able to modulate and prolong the half-life of the drug and potentially reduce its side effects, especially in the first hours.

The parameters and graph of the release kinetics study are shown in Table 3 and Figure 3B. Given the relationship obtained based on the percentage values of MB release as a function of time, the first-order model showed the best correlation coefficient, with R^2^ = 0.97. This model refers to a system, whose release rate is only a function of the remaining drug concentration, such as soluble active agents incorporated in a porous matrix, in which the amount of drug released is proportional to the amount of drug remaining in the matrix, which speaks directly with the polymeric nanocarrier developed in this study and the hydrophilic nature of MB [40].

Previous studies of MB encapsulation in PLGA nanoparticles have already reported this rapid release profile, such as Cannavà et al., who obtained total release of MB in 24 h in nanoparticles not associated with nonionic amphiphilic cyclodextrin (SC6OH). Working with the same polymer and combining single and double emulsification techniques, Gutiérrez-Valenzuela et al. obtained release above 80% of MB in the first 4 h for all tested formulations [19,41].

### 3.3. Generation of Reactive Oxygen Species

The generation of reactive oxygen species by methylene blue was studied by absorbance decay of DPBF. In ROS-rich environments, particularly in the presence of singlet oxygen, DBPF can quantitatively react with ^1^O_2_ to form o-dibenzoylbenzene, thus losing its ability to absorb or emit visible light [42]. Methylene blue is widely used in the literature as a standard photosensitizer to calculate the quantum yield of other substances, s its ROS production is sufficiently known. Still, in photodynamic therapy, the substance to be used as a photosensitizer must generate ROS in a satisfactory manner, but not in an exacerbated manner that could damage healthy cells [9,43,44]. Herein, a similar DPBF absorbance decay profile was observed between the MB solution and nanoparticle groups, in the first 3 min. The speed of ROS production by the nanoparticle in this period can be explained by the non-encapsulated MB. After this time, the nanoparticle shows a smaller influence on absorbance decay, which can infer a more gradual and less pronounced ROS generation than the solution, a result that is expected since the MB is encapsulated in a polymeric matrix. However, It should be noted that the encapsulation did not compromise the generation of ROS. At the end of 30 min of irradiation, the DPBF showed an absorbance of about 48.67% compared to the initial absorbance for the nanoparticle and 26.31% for the solution. Interestingly, to obtain the DPBF decay curve with the solution, it needed to be concentrated about 3 times, with a final concentration of 240 µg/mL, contrasting with the concentration of 80 µg/mL of the nanoparticle. This finding may reveal a greater potential of the nanoparticle, which, in addition to the controlled generation of ROS, requires a smaller amount of MB to act similarly to the solution. Seong and Jin (2015), working with MB encapsulated in calcium phosphate nanoparticles, obtained similar results, where the nanoparticle obtained a more controlled ROS generation compared to the solution when dispersed in N,N-dimethylformamide [35]. In another study, pegylated gold nanoshells containing methylene blue exhibited significantly higher ROS production than the MB solution [9].

### 3.4. In Vitro Photocytotoxicity and Cytotoxicity

**Figure 4:**
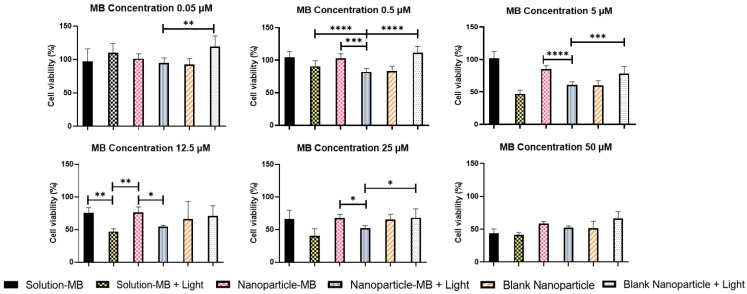
Cell viability of the optimized formulation, methylene blue solution and blank nanoparticle, with and without light, with an incubation time of 5 min. One-way ANOVA test with Bonferroni posttest between groups. * *p* < 0.05; ** *p* < 0.01; *** *p* < 0.001; **** *p* < 0.0001.

**Figure 5:**
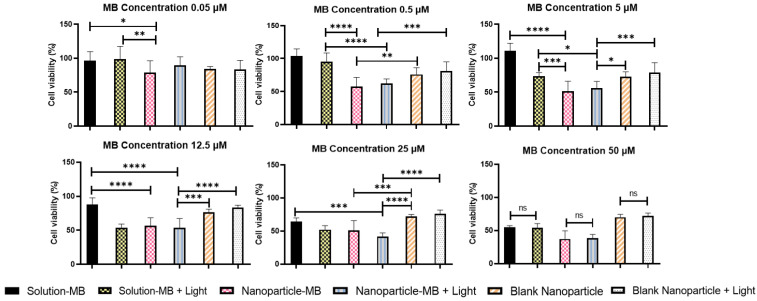
Cell viability of the optimized formulation, methylene blue solution and blank nanoparticle, with and without light, with an incubation time of 2 h. One-way ANOVA test with Bonferroni posttest between groups. * *p* < 0.05; ** *p* < 0.01; *** *p* < 0.001; **** *p* < 0.0001.

PDT is a less invasive technique for cancer therapy and can be enhanced if combined with the protection of the photosensitizing agent through nanoencapsulation. Figure 4 and Figure 5 demonstrate the respective cell viability of each formulation (solution-MB, nanoparticle-MB, and blank nanoparticle) at their respective concentrations and exposure or not to light in A431 cells. The photothermal effect related to light irradiation was considered negligible since the temperature during PDT application did not change.

Herein, three main aspects can be discussed—the contribution of encapsulation, the increase in incubation time, and the influence of light to increase MB cytotoxicity. First, note that cytotoxicity is concentration-dependent for all situations tested, and the statistically significant differences between solution and nanoparticle are evidenced mainly at intermediate concentrations of 0.5; 5; 12.5; 25 µM. In the incubation time of 5 min, a similar behavior of both groups tested without application of light is observed, with viability values of 104.21; 101.94; 75.56; 66.42% and 102.98; 85.40; 76.48; 67.50% for MB-solution and MB-nanoparticle, respectively, at the aforementioned concentrations. In the literature it is reported that the photosensitizer should not be toxic in the dark, consequently we can infer that the nanoencapsulation did not disturb the mechanisms of MB toxicity nor amplify them [14].

Exposure to light, on the other hand, increased the cytotoxicity of the groups, especially intensifying the MB-solution, reaching values of 90.87; 47.00; 46.96; 40.70% against 82.21; 60.77; 54.18; 51.76% of the nanoparticle. Still, when comparing the influence of light only in the group of nanoparticles, a statistically significant difference was found in all intermediate concentrations, the strongest being at 5 µM, where cell viability dropped from 85.40 to 60.77% (Figure 4). Although the effect of light was already expected by the trigger for the production of reactive oxygen species, the negative performance of the nanoparticle against the solution can be explained by its reduced time of contact with the cell. To evaluate this hypothesis, the tests were repeated simulating the previous conditions, changing only the incubation time of the treatments to 2 h [45].

Figure 5 shows the results with an incubation time of 2 h. The maintenance of the previously reported dose-dependent response is observed, along with the significant increase in cell death by exposure to light at all concentrations. However, in this incubation period, a relevant fact to be discussed is the less evident influence of light on the phototoxicity of the nanoparticle. It is known that when encapsulated, MB can be delivered more efficiently to its target, without suffering degradation or losing its photodynamic activity as it occurs in its free state, increasing its photocytotoxic effects. Furthermore, PS drugs have some dark toxicity, and this intensifies with increasing incubation time. This set of factors can help us understand the behavior and influence of light application along with the modulation of incubation time [46]. This can be demonstrated numerically in Table 4, when comparing the incubation time of 5 min and 2 h, IC_50_ suffers a sudden drop of approximately seven times with the application of light for the first one while in the last one this drop is only two times. Finally, the generation of reactive oxygen species must be the main mechanism of MB cytotoxicity. As demonstrated in topic 3.3, the nanoparticle generates ROS in a controlled and prolonged manner, which corroborates the result of greater cytotoxicity of the nanoparticle when we extended the incubation time to 2 h.

The result is in agreement with a study carried out by Gontijo et al., which demonstrated greater cytotoxicity in higher concentrations of MB, using the same cell lineage. However, in this case, the nanoparticles proved to be much more cytotoxic at all concentrations, while there was a worsening in the performance of the solution, which was statistically evidenced at concentrations of 0.5 and 5 µM, where cell viability reached values of 95.18 and 73.44% for the solution and 62.32 and 56.09% for the nanoparticle, respectively. The worse performance of the solution can be explained by a possible degradation and enzymatic reduction in the biological environment, making the photodynamic activity of free MB insignificant. This issue has already been addressed in a study where calcium phosphate nanoparticles were developed for enzymatic protection of MB (CaP-5MB). In that study, free MB and CaP-5MB reduction behaviors were confirmed by monitoring the absorption decrease at 663 nm. While, in the free MB solution, there was a decrease of approximately 100% in the intensity of absorption, CaP-5MB showed a minimal decrease over half an hour. In addition, it is also noted that the increase in incubation time favors the nanoparticle, as this stimulates cell uptake by the cell, causing the MB to act more effectively in the cytoplasm. Thus, the importance of encapsulation in protecting the photodynamic activity of MB is noted [27,35]. Finally, the blank nanoparticles were used as a control to evaluate the cytotoxicity of the nanostructure and groups of cells without any type of treatment used as a control of light toxicity. In both cases, the influence on cell viability was not of great magnitude with values ranging from 93.03 to 77.46%.

From the quantification of cell viability, it was possible to calculate the IC_50_ for the different groups (Table 4). The nanoparticle with light irradiation stands out with the best IC_50_ in the 2 h incubation period, being a possible choice to enhance PDT and decrease its side effects in treatments with long application times.

### 3.5. Cellular Uptake

**Figure 6:**
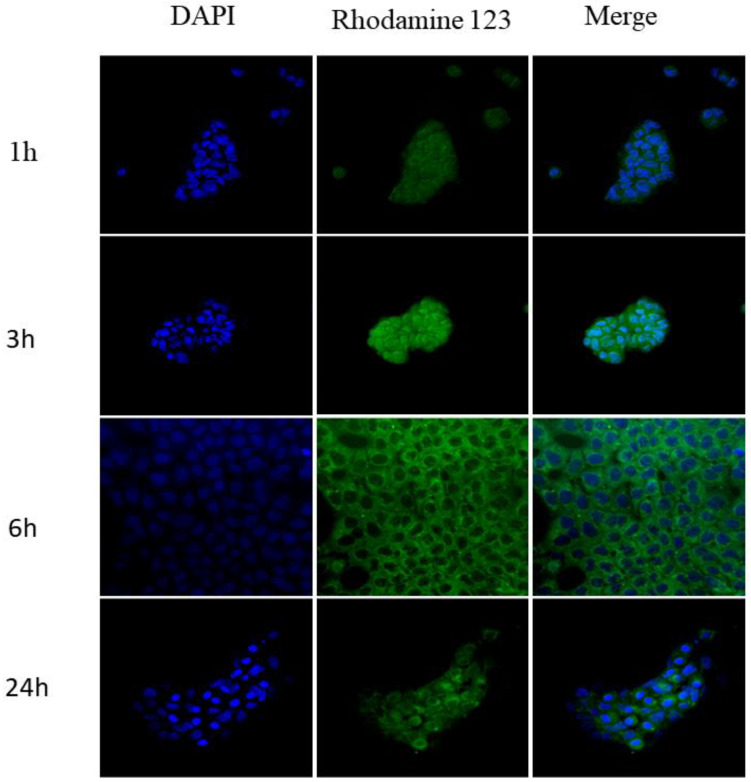
Cellular uptake of nanoparticles labeled with Rhodamine 123 and DAPI at times 1, 3, 6 and 24 h by confocal microscopy.

Nanoparticles labeled with Rhodamine 123 were incubated with A431 cells at times of 1, 3, 6 and 24 h. The results of confocal microscopy (Figure 6) showed increased cell uptake over time, with its peak between 3 and 6 h. This fact helps to explain the increase in photo/cytotoxicity observed previously, when the nanoparticles incubation time was increased to 2 h. Close to 24 h, a decrease in fluorescence can be seen, which may indicate degradation of the nanoparticle by the cell itself. In addition, fluorescent dots accumulate mainly in the cell cytoplasm, information that is in line with several studies in the literature in which the vast majority of nanoparticles with application in cancer therapy accumulate in the cytoplasm [18,47].

### 3.6. In Vitro Skin Penetration Studies

First, to quantify MB in the skin, UV-vis spectrophotometry was used. A spectral scan of skin samples with and without application of MB extracted in methanol was performed to verify whether the skin components interfered with the quantification. The results demonstrated that, in the region between 570 and 670 nm, there is no interference from the skin that could impair the quantification of MB, since proteins and other tissue components have their absorbance peak between 200 and 300 nm. In addition, in order to verify the sensitivity of the quantification technique, its recovery percentage was measured through contamination of the skin with known concentrations of the drug. The results showed an average of 96.92%.

It is known that topical formulations are limited by low skin permeability. Specifically, the stratum corneum (SC), the outermost layer of the skin, which acts as a barrier to the external environment. In this context, the resistivity measurement is an important parameter to infer the penetration of substances into the skin, since low values mean some structural alteration, facilitating this process [25,48]. Herein, the resistivity values obtained without the application of sonophoresis are close to 85.94 kΩ cm^2^, which indicates preservation of the skin structure. When using ultrasound as a pre-treatment, the resistivity dropped to approximately 1 kΩ cm^2^, indicating ultrastructural defects in the lipid regions of the stratum corneum.

**Figure 7:**
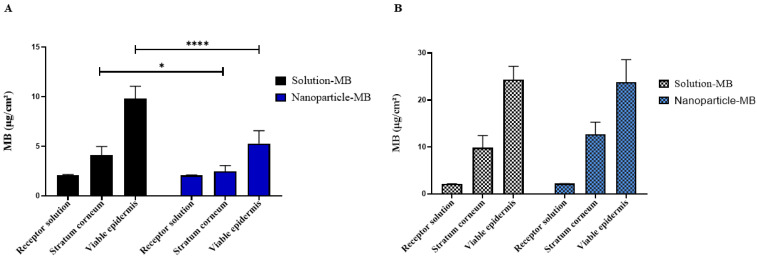
Graphical representation of MB quantification in solution and nanoparticle in stratum corneum (SC) and viable epidermis and dermis (EV + D) without (**A**) or with application of sonophoresis (**B**) in swine ear skin after penetration study by 16 h. * *p* < 0.05; **** *p* < 0.0001.

Figure 7 shows the quantification of MB in the stratum corneum and in the viable epidermis after 16h of study with passive penetration and application of sonophoresis. The amount of MB permeated in the receptor phase was below the quantification limit of the method, suggesting a very low transdermal transport. In the passive penetration of MB, there is a superiority of the solution in relation to the nanoparticle, both in the stratum corneum and in the viable epidermis, with values of 4.10 ± 0.87, 9.81 ± 1.24, 2.46 ± 0.59 and 5.27 ± 1.29 μg/cm^2^, respectively. It is known that the hydrophilic nature of this drug does not favor transport through the lipid CS, which ends up hampering skin penetration. Furthermore, the use of topical polymeric nanoparticles is less widespread than lipid systems such as liposomes, where penetration of intact polymeric nanoparticles through the stratum corneum barrier is unlikely [49,50,51].

Overall, sonophoresis is a physical method known to enhance drug delivery both topically and transdermally. However, the mechanisms promoted by ultrasound energy are still discussed in the literature. Cavitation, thermal effects and mechanisms related to convection seem to be the most common way to increase skin penetration [39]. Herein, when using it as a pre-treatment, the amount of MB reached higher levels of skin penetration, showing values of 9.85 ± 2.60, 24.30 ± 2.88 for the solution and 12.71 ± 2.55, 23.80 ± 4.77 μg/cm^2^ for the nanoparticle, in the stratum corneum and viable epidermis, respectively. With regard to nanoparticles, this increase was even more significant when compared to passive penetration. For example, for the solution group, MB skin penetration was, respectively, 9.81 ± 1.24 and 24.30 ± 2.88 μg/cm^2^ in the viable epidermis before and after the application of sonophoresis. By directly comparing these values, we obtain an increase in skin penetration of approximately 2.47 times. The nanoparticle, on the other hand, led to MB penetration of 5.27 ± 1.29 and 23.80 ± 4.77 μg/cm^2^, respectively in the viable epidermis before and after the application of sonophoresis, which means an increase of 4.51 times, which becomes interesting because it is in this layer that we can find SCC tumors. If we extend the analysis to the stratum corneum, we will see an even greater difference of 2.4 against 5.16 times for the same groups. Furthermore, an alternative to further improve the performance of the nanoparticle would be its incorporation into a gel matrix, thus increasing the contact time between the formulation and the skin. The skin penetration of the nanoparticle is similar to the solution when associated with sonophoresis, as shown in Figure 7B, without significant statistical difference.

Herein, the combination of sonophoresis and nanotechnology proved to be efficient. While the former causes alterations in the cutaneous structure, NPs, due to their greater surface area, accumulate in cutaneous appendages, such as hair follicles, which can bypass the formidable SC barrier. Currently, MB has obtained better penetration results when associated with nanocarriers, as in the study by Garcia et al., who, when developing liquid crystals and comparing the lamellar and hexagonal crystalline phases, obtained greater cutaneous delivery of MB with the phase lamellar, reaching values from 9.79 ± 1.22 to 15.70 ± 1.54 μg/cm^2^ in 6 h. Working with another known photosensitizer, the use of sonophoresis increased dermal penetration of zinc phthalocyanine, resulting in reduced viability of melanoma cells [29,52].

## 4. Conclusions

The Box–Behnken factorial design strategy proved to be an excellent and economically viable strategy for obtaining optimized formulations. Herein, we successfully developed and optimized PCL nanoparticles containing methylene blue, which achieved a high encapsulation efficiency, low polydispersion index and an optimal particle size, which are essential for a better skin penetration. The results obtained in scanning electron microscopy confirmed the formation of spherical nanostructures. The in vitro release of the nanoparticle demonstrated an initial burst profile compatible with the first-order model, which can be a positive feature for the simultaneous treatment of PDT + sonophoresis. Regarding the evaluation of cytotoxicity and its cellular uptake, the results achieved were promising, where A431 cell death was higher when combining the formulation with red LED light irradiation and the effects could be enhanced by increasing the incubation time. It was also shown that the possible mechanism of action of the MB nanoparticle is through the generation of oxygen species, in a controlled and gradual way. Furthermore, nanoparticle cell uptake increased with time and was higher between 3 and 6 h. We showed that the combination between nanoparticle and sonophoresis increased the cutaneous penetration of MB, and yet no significant amount was found in the receptor compartment, indicating that the formulation is only intended for topical application. Thus, we expect that MB nanoparticles will be equally or more efficient in the treatment of squamous cell carcinoma, which will be investigated in vivo in future studies.

## Figures and Tables

**Table 1:** Box–Behnken experimental design showing several runs and their relationships between independent factors X_1_, X_2_ and X_3_ with their actual values and respective dependent responses Y_1_, Y_2_ and Y_3_ of the prepared NPs, together with their zeta potentials.

N° of Formulations	X_1_ (Organic Phase/Aqueous Phase of the 1st Emulsification)	X_2_ (%PVA 1st Emulsification)	X_3_ (Amount of Polycaprolactone (mg))	Y_1_ Size (nm)	Y_2_ PDI	Y_3_ Encapsulation Efficiency (%)	Zeta Potential (mV)
1	3	0.5	45	278.06 ± 1.40	0.33 ± 0.01	96.05 ± 0.04	−4.19 ± 0.17
2	5	0.5	45	223.90 ± 1.72	0.15 ± 0.01	96.82 ± 0.03	−4.31 ± 0.20
3	3	1	45	252.33 ± 2.41	0.27 ± 0.01	94.93 ± 0.01	−3.80 ± 0.23
4	5	1	45	211.53 ± 1.24	0.17 ± 0.01	98.63 ± 0.11	−3.56 ± 0.21
5	3	0.75	30	262.76 ± 1.48	0.28 ± 0.01	95.63 ± 0.01	−3.20 ± 0.10
6	5	0.75	30	183.40 ± 1.10	0.17 ± 0.01	94.37 ± 0.03	−8.93 ± 0.10
7	3	0.75	60	271.83 ± 5.91	0.23 ± 0.02	98.59 ± 0.01	−11.96 ± 0.26
8	5	0.75	60	230.16 ± 1.50	0.11 ± 0.01	99.44 ± 0.07	−5.87 ± 0.15
9	4	0.5	30	234.96 ± 0.61	0.20 ± 0.01	95.19 ± 0.09	−5.15 ± 0.35
10	4	1	30	228.13 ± 2.65	0.12 ± 0.01	96.01 ± 0.02	−2.79 ± 0.06
11	4	0.5	60	260.20 ± 1.63	0.15 ± 0.01	95.26 ± 0.02	−2.50 ± 0.12
12	4	1	60	206.80 ± 2.35	0.12 ± 0.01	96.67 ± 0.31	−3.66 ± 0.11
13	4	0.75	45	212.63 ± 3.59	0.19 ± 0.01	97.85 ± 0.06	−3.23 ± 0.04
14	4	0.75	45	171.60 ± 2.19	0.11 ± 0.01	97.81 ± 0.02	−3.46 ± 0.38
15	4	0.75	45	160.43 ± 1.56	0.09 ± 0.02	96.04 ± 0.02	−4.28 ± 1.01

**Table 2:** Predicted values in optimization of the Minitab^®^ software and the actual values obtained for the optimized formulation of nanoparticles containing methylene blue.

Factor		Level Optimized	
X_1_: Organic phase/aqueous phase ratio 1st emulsification		4.59	
X_2_: Percentage in PVA (%) gives 1st emulsification		0.82	
X_3_: Amount in polycaprolactone (mg)		52.42	
Reply	Value estimated	Value obtained	Error relative (%)
Y_1_: Size in particle (nm)	186.76	156.93 ± 8.26	15.97
Y_2_: Polydispersion Index	0.11	0.11 ± 0.05	2.56
Y_3_: Efficiency in encapsulation (%)	98.39	94.22 ± 2.19	4.23

**Table 3:** Kinetic parameters of MB release from nanoparticle.

Mathematical Models	Zero Order		First Order		Higuchi		Weibull		Hopfenberg	
Parameter(s)	R^2^	0.75	R^2^	0.97	R^2^	0.85	R^2^	0.96	R^2^	0.94
	K_0_	10.04	K_1_	0.71	K_H_	34.29	α	0.83	$K_{HB}$	0.12
							β	0.60	n	3.38
							Ti	0.40		

**Table 4:** IC_50_ of the solution and nanoparticles containing MB with or without light irradiation in the incubation times of 5 min and 2 h.

Samples	IC_50_ (µM) Incubation Time: 5 min	IC_50_ (µM) Incubation Time: 2 h
Solution-MB	49.26	79.83
Solution-MB + Light irradiation	7.36	40.45
Nanoparticle-MB	213.79	22.37
Nanoparticle-MB + Light irradiation	26.81	9.90

## Data Availability

The data presented in this study are available on request from the corresponding author.
